# Healthy Country

**Published:** 1857-12

**Authors:** 


### A HEALTHY COUNTRY.

An observant clerical correspondent writes : "A fine old gentleman was invited to dine with me a few days ago, and remarked, that he knew thirteen persons in the circuit of a few miles, who had died within six months, and that all of them were over eighty years of age. Among these were:

| | Years. | | Years. |
|---|---|---|---|
| Nathaniel Raglan . . . | 82 | Nathaniel Haggard . . | 87 |
| Thomas Allen . . . . | 82 | John Garden. . . . . | 87 |
| Frederick Stipp . . . . | 82 | John Hedges . . . . | 87 |
| Mrs. Cunningham . . . | 86 | Mrs. Nichols. . . . . | 117 |

My informant was over eighty years of age. Among my hearers was a lady who was married in the year eighteen hundred, and yet had never before heard a sermon from a Presbyterian clergyman. From the above narration, we feel impelled to one of two conclusions, either Clarke County, Ky., must be a very healthy region, or that an entire ignorance of Presbyterianism is promotive of longevity. If our correspondent can ascertain that all these old soldiers had, in early life, enlisted under the banners of Presbytery, we will breathe freer.

